# Digital Interventions to Understand and Mitigate Stress Response: Protocol for Process and Content Evaluation of a Cohort Study

**DOI:** 10.2196/54180

**Published:** 2024-05-06

**Authors:** Josh Martin, Alice Rueda, Gyu Hee Lee, Vanessa K Tassone, Haley Park, Martin Ivanov, Benjamin C Darnell, Lindsay Beavers, Douglas M Campbell, Binh Nguyen, Andrei Torres, Hyejung Jung, Wendy Lou, Anthony Nazarov, Andrea Ashbaugh, Bill Kapralos, Brett Litz, Rakesh Jetly, Adam Dubrowski, Gillian Strudwick, Sridhar Krishnan, Venkat Bhat

**Affiliations:** 1 Interventional Psychiatry Program St Michael's Hospital Unity Health Toronto Toronto, ON Canada; 2 Department of Electrical, Computer, and Biomedical Engineering Toronto Metropolitan University Toronto, ON Canada; 3 Massachusetts Veterans Epidemiology Research and Information Center VA Boston Healthcare System Boston, MA United States; 4 Department of Psychiatry Boston University Chobanian and Avedisian School of Medicine Boston, MA United States; 5 Allan Waters Family Simulation Program Unity Health Toronto Toronto, ON Canada; 6 Department of Physical Therapy University of Toronto Toronto, ON Canada; 7 Neonatal Intensive Care Unit St Michael’s Hospital Unity Health Toronto Toronto, ON Canada; 8 Li Ka Shing Knowledge Institute St Michael’s Hospital Unity Health Toronto Toronto, ON Canada; 9 Department of Pediatrics Faculty of Medicine University of Toronto Toronto, ON Canada; 10 maxSIMhealth Group Ontario Tech University Oshawa, ON Canada; 11 Dalla Lana School of Public Health University of Toronto Toronto, ON Canada; 12 MacDonald Franklin OSI Research Centre Lawson Health Research Institute London, ON Canada; 13 School of Psychology University of Ottawa Ottawa, ON Canada; 14 Institute of Mental Health Research University of Ottawa Ottawa, ON Canada; 15 Centre For Addiction & Mental Health Toronto, ON Canada; 16 Institute of Health Policy, Management and Evaluation University of Toronto Toronto, ON Canada; 17 Arthur Labatt Family School of Nursing Western University London, ON Canada; 18 Department of Psychiatry University of Toronto Toronto, ON Canada

**Keywords:** web-based platform, stress, distress, moral distress, wearable, oura ring, virtual reality, VR, COVID-19, nursing, digital health implementation

## Abstract

**Background:**

Staffing and resource shortages, especially during the COVID-19 pandemic, have increased stress levels among health care workers. Many health care workers have reported feeling unable to maintain the quality of care expected within their profession, which, at times, may lead to moral distress and moral injury. Currently, interventions for moral distress and moral injury are limited.

**Objective:**

This study has the following aims: (1) to characterize and reduce stress and moral distress related to decision-making in morally complex situations using a virtual reality (VR) scenario and a didactic intervention; (2) to identify features contributing to mental health outcomes using wearable, physiological, and self-reported questionnaire data; and (3) to create a personal digital phenotype profile that characterizes stress and moral distress at the individual level.

**Methods:**

This will be a single cohort, pre- and posttest study of 100 nursing professionals in Ontario, Canada. Participants will undergo a VR simulation that requires them to make morally complex decisions related to patient care, which will be administered before and after an educational video on techniques to mitigate distress. During the VR session, participants will complete questionnaires measuring their distress and moral distress, and physiological data (electrocardiogram, electrodermal activity, plethysmography, and respiration) will be collected to assess their stress response. In a subsequent 12-week follow-up period, participants will complete regular assessments measuring clinical outcomes, including distress, moral distress, anxiety, depression, and loneliness. A wearable device will also be used to collect continuous data for 2 weeks before, throughout, and for 12 weeks after the VR session. A pre-post comparison will be conducted to analyze the effects of the VR intervention, and machine learning will be used to create a personal digital phenotype profile for each participant using the physiological, wearable, and self-reported data. Finally, thematic analysis of post-VR debriefing sessions and exit interviews will examine reoccurring codes and overarching themes expressed across participants’ experiences.

**Results:**

The study was funded in 2022 and received research ethics board approval in April 2023. The study is ongoing.

**Conclusions:**

It is expected that the VR scenario will elicit stress and moral distress. Additionally, the didactic intervention is anticipated to improve understanding of and decrease feelings of stress and moral distress. Models of digital phenotypes developed and integrated with wearables could allow for the prediction of risk and the assessment of treatment responses in individuals experiencing moral distress in real-time and naturalistic contexts. This paradigm could also be used in other populations prone to moral distress and injury, such as military and public safety personnel.

**Trial Registration:**

ClinicalTrials.gov NCT05923398; https://clinicaltrials.gov/study/NCT05923398

**International Registered Report Identifier (IRRID):**

DERR1-10.2196/54180

## Introduction

### Background

Work-related stress is a major issue encountered by health care workers (HCWs), as they face a variety of stressors due to the nature of their work, which often involves high-pressure situations and contact with pain and death [1]. The COVID-19 pandemic caused numerous difficulties for many individuals, with HCWs bearing unprecedented professional and personal demands due to challenges such as resource and staffing shortages. Nurses were affected most significantly, reporting the highest levels of burnout, emotional exhaustion, and psychological distress among all HCWs [2]. As COVID-19 cases decreased, levels of emotional exhaustion and psychological distress also decreased but continued to remain higher than the levels reported before the pandemic [2]. Prolonged elevated levels of stress can lead to anxiety, depression, and chronic diseases such as hypertension [3]. Work-related stress can also lead to burnout, a major contributor to nursing shortages faced by health care systems across the globe [4]. In a recent survey, 62% of Canadian nurses said they were considering leaving nursing altogether, citing stressors such as increased workload, staffing shortages, and frequent overtime [5]. Importantly, excessive stress can also impact the quality of care; increased stress is associated with increased medical errors [6] and decreased caring behaviors [7].

Continued unrelenting personal and work-related challenges have, at times, resulted in individuals compromising their beliefs and values to make difficult decisions, which may have life-or-death consequences [8-12]. Clinicians have reported acting in ways that are contrary to their moral values, integrity, and professional commitments, particularly during the initial phases of the COVID-19 pandemic [13,14]. Moral suffering can ensue when a clinician’s moral foundation has been threatened or violated because of witnessing, participating in, or precipitating decisions or actions that degrade their integrity [15].

Moral suffering encompasses the related but distinct concepts of moral distress and moral injury. Moral distress was first described within the nursing context by Jameton [16] as “...when one knows the right thing to do, but institutional constraints make it nearly impossible to pursue the right course of action.” For example, commonly cited sources of moral distress in nursing include inadequate staffing, inadequate pain relief for patients, and prolongation of life support when they believe it is not in the best interest of the patient [17]. First defined within the military and veteran context, moral injury was said to be the maladaptive biopsychosocial and spiritual outcome of a betrayal of moral character, typically by an individual holding authority [18]. Since this original definition was suggested, literature within the military context has expanded the definition to include maladaptive outcomes following perpetration, witnessing, and being the victim of acts that violate one’s moral values [19]. Importantly, moral suffering is thought to exist on a continuum, with moral injury representing a more extreme outcome; repeated instances of moral distress can ultimately lead to moral injury, which is characterized by impaired function and longer-term psychological harm [20]. Also of relevance is the concept of potentially morally injurious events, which have been described in nursing as situations in which professionals find it impossible to carry out the usual standards of care [21].

Limited information exists on treating and preventing moral injury and moral distress, especially in a health care context. While moral injury may have overlapping symptoms with posttraumatic stress disorder (PTSD), treatments used for PTSD have generally been found to be less effective for the treatment of moral injury [22]. Effective treatments for moral injury are likely required to tap into integrity that is degraded, as strengthening an individual’s moral compass has been shown to be an effective way to promote healing [23]. Additionally, psychoeducation on ethics [24] and moral distress [25], support seeking [20], and engagement in critical self-reflection [26] have been suggested as potential interventions for moral distress.

Given that it is infeasible and unethical to subject HCWs to real-life events that evoke moral suffering, virtual reality (VR) represents a promising tool for studying this topic. Exposure to VR scenarios is more ethical, as it is unlikely to cause severe distress but has nonetheless been shown to elicit real psychophysiological responses [27,28]. Moreover, VR allows for the creation of customizable environments and real-time data capture of physiological responses in a controlled setting. VR has previously shown success in the assessment and treatment of a variety of mental health disorders, including anxiety-related disorders, schizophrenia, substance use disorders, and eating disorders [28]. In the assessment of mental health disorders, VR can be used to present potentially triggering scenarios during which physiological measurements are made. For example, several studies have used VR to simulate combat situations while measuring markers of stress in those with PTSD [29-31]. Moreover, the ability of VR to simulate reality in a low-stakes environment renders it useful for exposure therapy. Indeed, a meta-analysis of studies that used VR exposure therapy (VRET) found that VRET was effective in treating a variety of anxiety and related disorders, including PTSD, and did not differ in effectiveness from in vivo exposure therapy [32]. Given the similarities previously outlined between moral injury and PTSD, and the variety of anxiety-related conditions that have been shown to be responsive to VRET, VR may be a promising tool for the treatment of stress and moral suffering.

In phase 1 of this project, we examined the feasibility of using our digital suite (VR and app-based monitoring of participants’ physical activity and subjective states) in 15 HCWs to understand stress and moral distress. This work was published as a protocol paper [33], a machine learning model used to predict moral distress [34], and a summary of our results [35]. Based on the feasibility demonstrated in phase 1, we are completing phase 2 with several key changes. First, the study will be conducted with a larger sample of 100 nursing professionals to evaluate the preliminary efficacy of our digital suite in mitigating and understanding both stress in general and moral distress in particular. Additionally, using feedback from phase 1, we developed a new VR simulation and educational video, as described by Sivanathan et al [36]. We aimed to create a more immersive environment to increase perspective-taking and heighten moral awareness, closing the dissonance in moral judgment between hypothetical scenarios and real-life situations. Also new in this study, we will use a wearable device [37] to longitudinally monitor participants’ physiological states. Combining this with longitudinal monitoring of participants’ subjective states with a web-based platform, we will use a novel machine learning approach [38] to predict stress and moral distress on an individual level. Furthermore, based on poor engagement in phase 1 [35], we have created a new strategy for encouraging participant compliance. Both phases 1 and 2 of the study are funded by the Canadian Department of National Defence.

### Objectives

In the current trial, nursing professionals (registered nurses and registered practical nurses) in the province of Ontario will be recruited. During the experimental visit, participants will use our digital intervention suite, composed of (1) a VR moral decision-making simulation and educational video, to examine the acute effects of the simulated moral dilemmas. For at least 2 weeks before and 12 weeks after the intervention, participants will complete questionnaires through (2) a web-based platform and (3) use a wearable device to longitudinally monitor the effects of the VR session.

In conducting this trial, we have the following aims: aim 1—we will use a VR simulation to characterize and reduce stress and moral distress related to difficult decision-making in complex moral situations faced by nursing professionals; aim 2—we will use a web-based platform to measure stress, moral distress, and other mental health symptoms, as well as a commercial wearable device to collect physiological data (this will be done to understand and examine the contribution of active, ie, subjective experiences as reported through self-report assessments, and passive, ie, physiological measurements collected without conscious participant input, data in predicting stress and moral distress); aim 3—we will create a personal digital phenotype profile (pDPP) based on the physiological data (ie, electrocardiogram, electrodermal activity (EDA), plethysmography, and respiration) collected during exposure to a hypothetical moral dilemma in a virtual environment, as well as the wearable and web-based data, to help understand its impact on stress and moral distress at the individual level.

## Methods

### Participants

A total of 100 nursing professionals from Ontario will be recruited to determine the preliminary efficacy of our digital intervention suite. Eligible participants must (1) be a registered practical nurse or a registered nurse currently employed at an Ontario health care institution and (2) be an owner of a smartphone. Exclusion criteria include (1) history of seizures except febrile seizures (as seizures are listed as an unlikely but possible symptom of using the VR headset [39]); (2) use of electronic medical devices (due to the risk of interference with physiological data collection); (3) score of ≥15 on the 7-item Generalized Anxiety Disorder-7 (GAD-7) [40] scale, an indication of severe anxiety; and (4) score of ≥20 on the 9-item Patient Health Questionnaire (PHQ-9) [41], an indication of severe depression.

Participants will be recruited through (1) social media advertisements, (2) flyers posted at St. Michael’s Hospital, and (3) email notices sent through various listservs (eg, the Canadian Nursing Informatics Association and the Nursing Research Interest Group). Interested participants will contact the study coordinator, who will send potential participants an informed consent form, a demographic form, and the PHQ-9 and GAD-7 scales. The coordinator will also confirm the individuals’ employment and lack of seizure history and electronic medical devices. If the eligibility criteria (as defined above) are met and the participant consents, they will be scheduled for a baseline visit at the interventional psychiatry program.

### Trial Design

**Figure 1:**
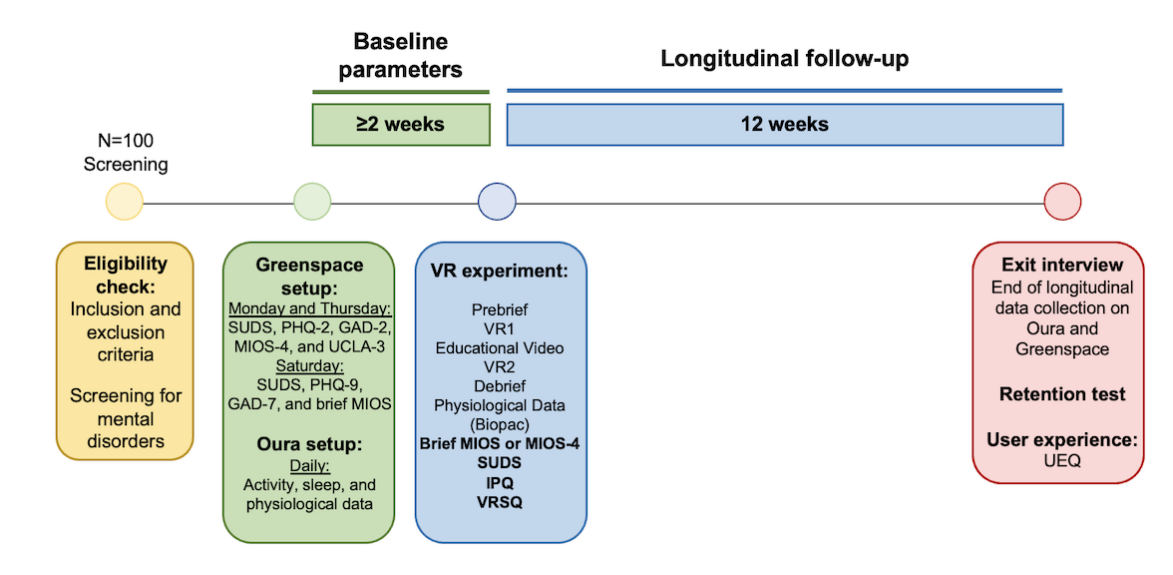
Phase 2 study schema. Brief MIOS: (clinical version) MIOS-4; GAD-2/7: Generalized Anxiety Disorder-2/7 item scale; IPQ: Igroup Presence Questionnaire; MIOS-4: Moral Injury Outcomes Scale; PHQ-2/9: Patient Health Questionnaire-2/9 item scale; SUDS: Subjective Units of Distress Scale; UCLA-3: UCLA 3-item loneliness scale; UEQ: User Experience Questionnaire; VR1: Virtual Reality Simulation Run 1; VR2: Virtual Reality Simulation Run 2; VRSQ: Virtual Reality Sickness Questionnaire Scale.

The trial will take place at St. Michael’s Hospital, Unity Health Toronto, Ontario, and will be composed of 3 main phases: baseline, VR simulation, and follow-up, which are visualized in Figure 1. The baseline period will last for a minimum of 2 weeks, during which participants will use the wearable every day and complete scales on a commercial web-based data collection platform, according to the schedule outlined in Figure 1. Following this, participants will engage in a VR simulation that requires complex moral decision-making. The simulation will be followed by an educational video on stress response, and then participants will repeat the simulation to provide them with the opportunity to apply the stress management strategies from the video. At the conclusion of the VR session, participants will engage in a semistructured debrief interview. Physiological signals will be collected through the entirety of the VR session and the debrief. The wearable and web-based platform will continue to be used by participants in the follow-up period, which will take place approximately 12 weeks following the VR session. As described in “Engagement Strategy” section, research team members will be actively involved during this follow-up period to encourage compliance and assess engagement. A semistructured exit interview will take place at the conclusion of participation.

### Data Storage

Eligible participants who are screened will be assigned a unique 7-digit ID. All identifiable information will be handled in one password-encrypted master linking file on the Unity Health Toronto server. The master linking file will contain identifiable information corresponding to the assigned participant’s unique ID. Deidentified information will be stored on 2 password-protected (through advanced encryption standard encryption) external hard drives located in a locked office at a Unity Health Toronto research site. Participant information will be deidentified on the wearable app and the web-based platform through the use of a unique ID and dummy emails. Data from each of these sources will be downloaded in a weekly data pull, and data from VR sessions will be transferred daily onto the external hard drives.

### VR Session

#### Design of VR Simulation

Details on the development of the simulation are described elsewhere [36]. Briefly, the content of the VR simulation was developed by a panel of subject-matter experts using a modified Delphi methodology [42]. The script was then created in an iterative manner, with versions reviewed and adjustments made on a regular basis until a final version was approved.

The VR simulation places a novice nurse (the research participant) in a high-stakes scenario within a general ward unit. Faced with a code-blue situation, the participant must make a critical decision between attending to one of 2 patients: one experiencing ventricular fibrillation and the other in cardiac arrest. Compounding the urgency, the general ward unit is devoid of additional medical staff and lacks emergency crash carts. On the second run-through of the VR simulation, participants are automatically directed to care for the same patient they chose during their initial experience. The design, software, and hardware are identical to phase 1 (reported in the protocol paper [33]).

**Figure 2:**
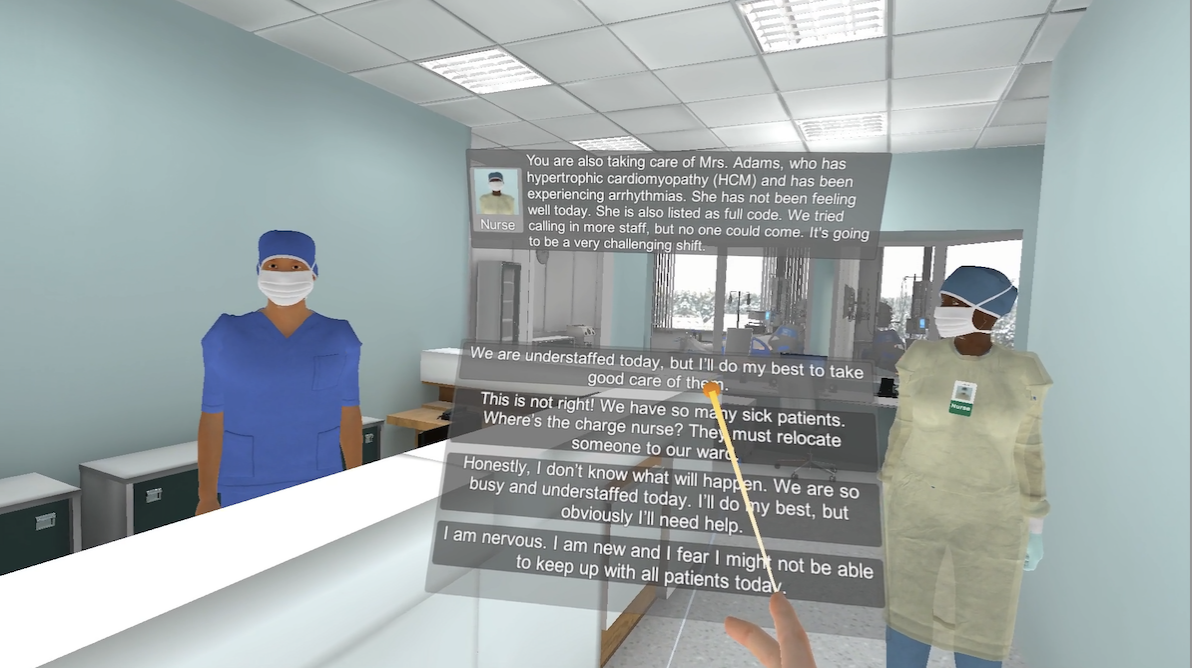
User interface displaying an example of a participant response.

At several points during the VR simulation, participants will be asked to respond to the situation by selecting a response from a list of options (Figure 2). Participants will be instructed before the VR simulation to select the responses that best represent their internal feelings, even if they are not reflective of what they would say in a professional setting. The research team will record all responses.

Participants will view the educational video (Multimedia Appendix 1) following the first simulation. The video was designed using a similar iterative feedback method and provides psychoeducation on stress and moral distress, as well as strategies to mitigate their effects. Specifically, we will teach unburdening techniques [43], self-compassion [44], and grounding tools (ie, diaphragmatic breathing [45]).

#### VR Data Collection

**Figure 3:**
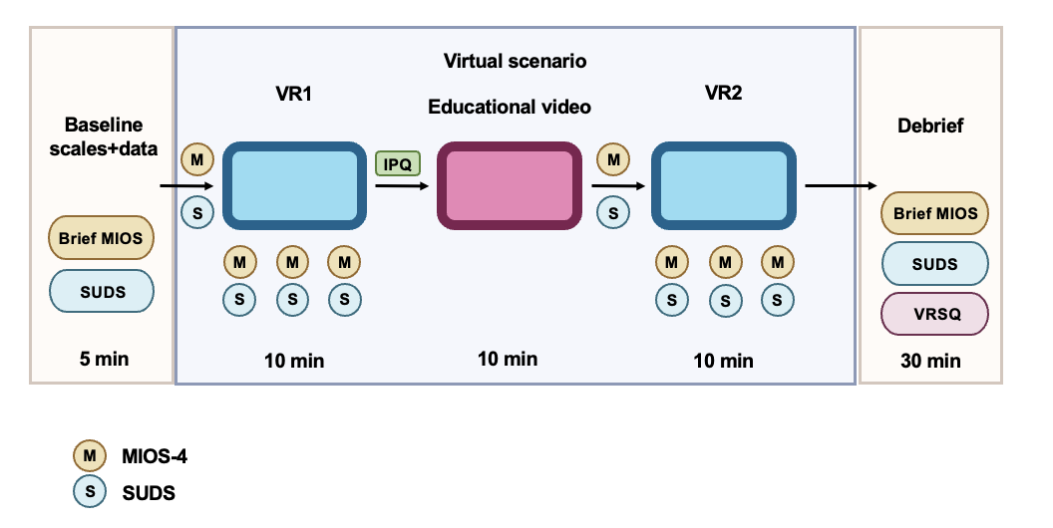
Design of the virtual reality (VR) experimental session. All scales during the VR session will be completed in the immersive virtual environment. Brief MIOS: (clinical version) MIOS-4; IPQ: Igroup Presence Questionnaire; MIOS-4: Moral Injury Outcomes Scale; SUDS: Subjective Units of Distress Scale; VR1: Virtual Reality Simulation Run 1; VR2: Virtual Reality Simulation Run 2; VRSQ: Virtual Reality Sickness Questionnaire Scale.

A visual overview of the data collection process during the VR simulation is shown in Figure 3.

A total of 4 scales and one short-form scale will be used to collect self-reported data during the session. The Moral Injury Outcome Scale (MIOS) [46] is used to assess an individual’s reaction to a potentially morally injurious event and is indexed to the most morally distressing experience of their life that currently distresses them. While the VR simulation is intended to simulate moral distress, the MIOS is designed to assess moral injury and, as such, is used as a proxy of our intended measure. This study will use the clinical version of the brief MIOS (Multimedia Appendix 2), which consists of 14 questions that assess an individual’s thoughts and feelings in response to a distressing event and eight items that assess how the event has impacted their daily life. The scale was adapted for nursing professionals by the research team in collaboration with the creator of the scale. Additionally, we will use a shortened version, termed the MIOS-4 (Multimedia Appendix 2), which contains the 4 items from the MIOS with the largest variance. The purpose of the MIOS during the VR session is 2-fold: first, it is used to characterize preexisting moral injury symptoms in participants. Second, the content of the scale is leveraged to assess acute reactions to the simulated moral dilemma.

The Subjective Units of Distress Scale (SUDS) [47] measures the level of distress an individual is experiencing at the moment. Individuals rate their current level of anxiety or discomfort on a scale of 0 to 100, in intervals of 10. The purpose of administering this scale is to obtain a general measure of acute distress elicited by exposure to a moral dilemma.

The Igroup Presence Questionnaire Scale [48] measures an individual’s sense of presence and reality during the VR session. This scale is used to assess the VR quality and user experience.

The Virtual Reality Sickness Questionnaire [49] assesses common side effects after VR exposure. This scale will be used to assess the safety and tolerability of the VR simulation.

**Figure 4:**
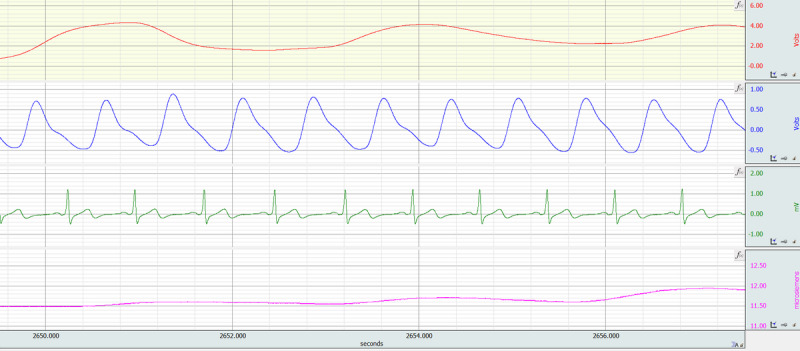
Physiological data collected during the virtual reality (VR) session and debrief. The image shows representative tracings of (from top to bottom): respiratory impedance (RI), photoplethysmography, electrocardiogram, and electrodermal activity (EDA) signals.

Physiological data will be collected throughout the duration of the VR session, from the beginning of baseline until the end of the debriefing. Data collection will be done using a Biopac MP160 (Biopac Systems Inc) system, as described in phase 1 [33]. The following 4 physiological signals will be collected as indicators of stress: EDA, electrocardiogram, respiratory impedance (RI), and photoplethysmography. EDA is a measure of the change in conductivity of the skin, which occurs due to changes in the activity of sweat glands. Since sweat gland activity is mediated by activation of the sympathetic nervous system, EDA provides a measure of sympathetic activity and, thus, arousal [50]. An electrocardiogram measures the electrical activity of the heart. Relevant features extracted from the electrocardiogram signal will include heart rate, which increases with arousal, and heart rate variability (HRV), a signal that reflects the activity of the autonomic nervous system and can be used as an indicator of stress [51]. RI, which is defined as the mechanical load of ventilation, will be used to measure the respiratory rate, which also increases with arousal [52]. Lastly, photoplethysmography uses infrared light to measure changes in blood volume. Similar to HRV, peripheral blood flow is mediated by the autonomic nervous system and, thus, is an indicator of acute mental stress [53]. Representative tracings of these signals are shown in Figure 4.

### Wearable

Passive data will be collected using an Oura Ring (Oura Health Oy), a commercially available wearable device [37]. The ring can be worn on the finger with minimal interference to collect a variety of physiological signals, including heart rate, workout heart rate, respiratory rate, HRV, daily activity, activity score, body temperature (delta), blood oxygen saturation, recovery score, sleep efficiency, and sleep score. The device will be worn for a minimum of 2 weeks before the VR session to ensure appropriate baseline data collection and will be worn continuously for 12 weeks post-VR. Participants will be asked to open the app daily to allow their data to be uploaded, and they can use the app to monitor their personal data.

### Web-Based Platform

We will use the web-based platform developed by Greenspace Mental Health (Greenspace) to collect active data during the trial [54]. This platform facilitates the continuous measurement of mental health symptoms and well-being through the regular administration of standardized assessments. Participants will complete these assessments for the duration of their enrollment in the trial, including the baseline period leading up to the VR session and the 12-week follow-up thereafter.

### Additional Scales

In addition to the brief MIOS or MIOS-4 and the SUDS, the scales in the following sections will be included on the web-based platform.

#### GAD Scale

The GAD Scale [40] is a measure that assesses the level of anxiety a person has experienced over the last 2 weeks. The scale consists of 7 items, with each item scoring on a Likert scale of 0 to 3. The total score ranges from 0 to 21, with higher scores indicating greater anxiety. The GAD-2 only includes items 1 and 2 of the GAD-7 and has a total score that ranges from 0 to 6.

#### PHQ Scale

The PHQ [41] is a self-report questionnaire assessing symptoms of depression over the last 2 weeks. The PHQ-9 scale consists of 9 items, with each item scoring on a 4-point Likert scale from 0 (not at all) to 3 (nearly every day). The total score ranges from 0 to 27, with higher scores indicating more severe symptoms of depression. The PHQ-2 includes only the first 2 items of the PHQ-9 and has a total score range of 0 to 6.

#### UCLA 3-item Loneliness Scale

The UCLA 3-item Loneliness Scale (UCLA-3) [55] is a self-report questionnaire assessing subjective feelings of loneliness and social isolation. This scale consists of three items, with each item scoring from 1 (hardly ever) to 3 (often). The total score ranges from 3 to 9, with higher scores indicating more severe symptoms of loneliness.

Scales will be administered 3 times per week, according to the schedule shown in Table 1.

**Table 1:** Scale administration schedule.

Day	Scale
Monday	SUDS^a^ GAD-2^b^ PHQ-2^c^ MIOS-4^d^ UCLA-3^e^
Thursday	SUDS GAD-2 PHQ-2 MIOS-4 UCLA-3
Saturday	SUDS GAD-7^f^ PHQ-9^g^ Brief MIOS^h^

^a^SUDS: Subjective Units of Distress Scale.

^b^GAD-2: Generalized Anxiety Disorder-2.

^c^PHQ-2: Patient Health Questionnaire-2.

^d^MIOS-4: Moral Injury Outcome Scale-4.

^e^UCLA-3: UCLA 3-item Loneliness Scale.

^f^GAD-7: Generalized Anxiety Disorder-7.

^g^PHQ-9: Patient Health Questionnaire-9.

^h^Brief MIOS: Brief Moral Injury Outcome Scale.

### Engagement Strategy

We will use several new strategies to maximize participant engagement. First, we will send daily SMS text messages reminding participants to use their wearable and to open the wearable app. The text messages will also include a reminder on Mondays, Thursdays, and Saturdays to complete the questionnaires. Second, we will send emails reminding participants on Mondays, Thursdays, and Saturdays to complete the questionnaires. Third, we will actively monitor which participants are missing data and contact those who are on a weekly basis. Different actions will be taken depending on the amount of missing data, which are outlined in Table 2.

Lastly, we will establish a compensation system to further encourage compliance. For every 2-week period following their VR session, participants will be compensated CAD $30 (US $21.93) if they did not go a single day without any missing active or passive data. Participants will be given this honorarium upon completing the study. Participants will also be given CAD $80 (US $58.48) for completing the screening visit, CAD $80 for the VR visit, and CAD $30 for the exit interview (see Exit Interview section).

**Table 2:** Engagement action according to the amount of missing questionnaire and wearable data received.

Severity level	Amount of missing data	Engagement action
Minor	Missing at least one day of questionnaires or one day of wearable data in a week.	Call participant and ask them to contact the research team if they are having technical difficulties.
Medium	Missing one or 2 days of questionnaires, 2 weeks in a row; 2 questionnaires in one week; or wearable data 3 days a week, 2 weeks in a row.	Call and text participants, ask them to contact the research team regarding their missing data regardless of whether there are no technical difficulties. Repeat contact daily until response is established.
Major	Missing questionnaire 3 days in a week; wearable 4 days or more in a week; or at least 3 days of wearable data in addition to one day of questionnaires per week.	Call and text participants, ask them to contact the research team regarding their missing data regardless of whether there are no technical difficulties. Repeat contact daily until response is established.

### Debrief

The debrief, which will take place immediately after the VR session, will be composed of a semistructured interview, open-ended feedback, and 3 Likert-rated statements (an interview guide is included in Multimedia Appendix 3). The semistructured interview will ask participants to reflect on and explore emotions elicited by the VR simulation and will test participants’ retention of the material from the educational video. Open-ended feedback and Likert-rated statements will explore participants’ impressions of the VR simulation and how it could be improved and made more realistic.

### Exit Interview

At the end of the 12-week follow-up period, participants will complete a semistructured exit interview regarding their experience in the study, including perceptions of the utility and relevance of the educational video and barriers to the use of digital platforms (an interview guide is included in Multimedia Appendix 4). Included in this interview will be a retention test on the concepts and strategies discussed in the educational video. After the interview, the participant will complete the User Experience Questionnaire for the wearable and web-based platform. The User Experience Questionnaire is a widely used questionnaire for measuring impressions of the user experience of interactive products [56,57].

### Quantitative Data Analysis

#### Sample Size

This project aims to demonstrate the preliminary utility of multimodal digital platforms to understand and mitigate general stress and moral distress. Sample size calculations were estimated based on the results of a post hoc analysis of a pilot study [33].

We calculated sample sizes for a 2-tailed paired *t* test to compare the means of the prescore and postscore of MIOS using the POWER procedure in SAS (version 9.4; SAS Institute). Parameters for the sample size calculation were estimated from a pilot study, as the SD of each score was 9.86 and the correlation of pre- and postscores was 0.75. A sample size of 55 pairs (55 patients) was estimated with a 2-sided α of 5% and a power of 90%. We set the effect size (mean difference between prescore and postscore) to detect at 3.13. Therefore, assuming a conservative dropout rate of 30%, the proposed sample size of 100 patients will still provide 70 pairs, which is sufficient to achieve >90% power based on the parameter assumptions.

#### Statistical Analysis

R (R Core Team) and SAS will be used to conduct statistical analysis. General participant characteristics and demographics will be listed in a baseline characteristics table. Continuous variables will be presented with the mean and SD when normally distributed, or with the median and IQR for skewed data. Dichotomous and categorical variables will be presented with frequency and percentage.

For the brief MIOS and other scales from the VR session (eg, MIOS-4 and SUDS), the prescore, postscore, and their differences will be summarized using descriptive statistics such as mean (SD) and median (IQR). For each scale, the prescore and the postscore will be compared using 2-tailed paired *t* tests. Similar analyses will be performed for the scales in the web-based platform (eg, GAD-7, GAD-2, PHQ-9, PHQ-2, and UCLA-3). The normality of all scale data will be assessed using a Shapiro-Wilk test to validate the use of the 2-tailed paired *t* test, which assumes normality. If the normality test indicates a significant deviation from a normal distribution, the Wilcoxon signed rank test, a nonparametric test for paired data, will be used. Results will be considered significant with a *P* value ≤0.05.

### Personal Digital Phenotype Profile

With the active and passive data collected throughout this study, we will perform digital phenotyping as outlined in aim 3 to further understand and quantify the experience of stress and moral distress in a naturalistic setting (details on the methodology are described by Nguyen et al [34]). Digital phenotyping is defined as the “moment-by-moment quantification of the individual-level human phenotype in situ using data from smartphones and other personal digital devices” [58]. This approach can be used to effectively represent longitudinal data and may present an opportunity for tailored monitoring of treatment response and relapse prediction in individuals with mental disorders [59]. Additionally, digital phenotyping allows for the monitoring of patients in natural contexts and in real time [59].

Once the digital phenotype profile (DPP) representation has been created, we additionally enhance this definition by proposing the term personal DPP (pDPP) as a personalized version of the DPP, where we collect longitudinal data to design digital biomarkers to monitor the changes in physical and behavioral health of an individual participant. The pDPP will be used to provide insight into a person’s affective state using an individual’s physiological data, even without any active data.

The process of digital phenotyping involves building a machine learning model using the active and passive data collected from each participant. An individual model is built for each person by incorporating their physiological data together with their self-reported affective states collected from questionnaires. In the long run, such models could allow for insights into nurses’ mental well-being in a nonintrusive way that can improve mental health care and responses to stress.

**Figure 5:**
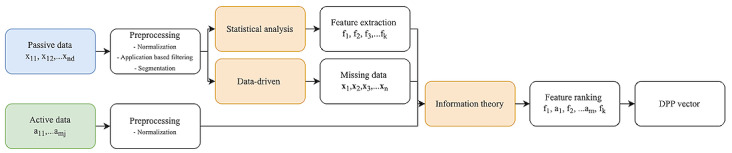
A block diagram representation of the proposed statistics, information theory, and data-driven (SID) pipeline (adapted from Nguyen et al [38], with permission from the Institute of Electrical and Electronics Engineers [IEEE]). DPP: digital phenotype profile.

Our goal is to perform a systematic investigation of the DPP with an emphasis on robustness for long-term utility. We envision the DPP to be represented as a multidimensional vector composed of passive and active data, where each profile is unique to one another. To do so, we propose a statistics, information theory, and data-driven (SID) pipeline to develop the foundation of the DPP as described by Nguyen et al [38]. The proposed novel SID pipeline is shown in Figure 5.

The DPP will be created through the use of the SID and will be a representation for each individual user. Once the DPP is created, we will enhance the analysis by proposing the pDPP, where we develop individual models for each participant by incorporating their physiological data together with their self-reported affective states collected from questionnaires. The pDPP will be used for long-term utility, where we monitor the changes in physical and behavioral health of an individual participant.

MATLAB (Cleve Moler) and Python (Python Software Foundation) language will be used for signal processing and machine learning. The physiological signals will be collected in raw format during the VR intervention and will be further sent to preprocessing, feature extraction, and machine learning techniques for data analysis. Preprocessing techniques involve filtering and cleaning the signal. Techniques such as low-pass, high-pass, and notch filters will be used to remove noise, and the resulting filtered signal will be submitted to the extraction of relevant features. Feature extraction will involve extracting attributes of the signal to be better represented. Both handcrafted features and deep learning features will be extracted. Techniques for feature extraction include handcrafted features and automatic deep learning features. Handcrafted features will involve statistical values extracted from the signal, whereas automatic deep learning features will be extracted through deep learning models. Since deep learning models will be used, the features may be abstract and better represent the data in a higher dimension. Lastly, machine learning techniques will be used for the final data analytics. Trends, classifications, and relationships will be determined using different machine learning techniques. Implementations of preprocessing, feature extraction, and machine learning will be conducted in MATLAB and Python.

Preprocessing, feature extraction, and machine learning techniques will be applied to the data collected during the VR intervention to identify data patterns and develop prediction models for stress and moral distress. Similarly, active data from the web-based platform and passive data from the wearable device will be analyzed using data-driven techniques to monitor participants’ activity and mental well-being in naturalistic environments and develop prediction models for stress and moral distress.

### Qualitative Analysis

Audio recordings from the post-VR debrief and exit interviews will be transcribed using a 3-stage approach. First, each audio file will be transcribed (audio to text) using an automated pipeline. Each transcript will then be reviewed for accuracy and adapted to simple transcription conventions that preserve conversational prosody and deidentify personally identifying information [60].

Thematic analysis will be used to extract recurring codes and overarching themes elicited during the post-VR debrief session and exit interview [61,62]. In particular, the post-VR debrief analysis will focus on themes arising from the emotional experiences elicited by the VR simulation, decisions made in the VR simulation, and feedback on how participants thought the scenario could be improved. The exit interview analyses will focus on themes emerging from participants’ recollection and impressions of the VR simulation, the perceived usefulness of the techniques presented in the educational video, and experiences with the wearable and web-based platform.

### Ethical Considerations

This study was approved by the St. Michael’s Hospital Research Ethics Board (22-279) in April 2023 before any participants were recruited.

## Results

This project was funded by Innovation for Defence Excellence and Security: Competitive Projects, Department of Defence, Canada in January 2022. The first participant was enrolled in May 2023. As of September 2023, all 100 participants had completed screening, and 99 had completed their VR session (1 dropout). The data collection was completed in December 2023.

## Discussion

### Potential Benefits

Participants may benefit from psychoeducation related to stress, moral distress, and techniques to address those experiences. Participants may also experience some degree of relief from moral distress throughout this study from the educational video, and the opportunity to better understand their mental and physical health through self-monitoring built into the web-based and wearable platforms. Further, participation in this study can benefit nursing professionals as a whole, contributing to the understanding and prevention of stress and moral distress in health care professions.

### Potential Risks

There are no serious risks associated with participation in this study. Participants may experience side effects associated with VR, such as nausea and dizziness, in which case the experiment will be paused. These symptoms are typically transient in nature and are expected to pass within hours after VR exposure. Participants will be asked about the occurrence of such symptoms during their VR session through the Virtual Reality Sickness Questionnaire scale. There is always a risk of data leaks when using digital platforms. However, data shared with third parties (ie, Greenspace and Oura) will be shared under a dummy email. As such, third-party platforms will have no identifiable information about participants. Data stored at St Michael’s Hospital, Unity Health Toronto will be stored on encrypted and password-protected drives. It is possible that exposure to a simulated distressing event may provoke negative feelings in participants, which warrants professional assistance. The study psychiatrist will be on call should such a situation arise. Finally, all adverse events will be recorded in an adverse event log maintained by study staff.

### Limitations

There are several limitations to this study. One drawback is the potentially limited ability of the VR simulation to mimic a real-life moral dilemma, which would lead to a less ecologically valid understanding of moral distress. Previous research using VR simulations has demonstrated the elicitation of genuine subjective experiences, including fear, anxiety, and paranoia, as well as objective psychophysiological responses such as changes in heart rate and skin conductance [27,28]. However, the VR simulation in phase 1 of this study induced limited changes in MIOS and Perceived Stress Scale scores [63], albeit in a small sample. Another limitation is the lack of a control group. Given that all 100 participants will be subjected to the VR simulation twice with the educational video played in between, we will not be able to discern whether changes in responses between the first and second instances of the VR simulation are caused by the educational video itself or other factors such as simulation repetition. That being said, evaluation of the efficacy of the educational video in reducing acute symptoms of stress is not the primary aim of this study.

### Future Directions

The proposed project could allow for the development of evidence-based methods for the prevention and treatment of stress, moral distress, and moral injury. This will allow for a conceptual integration of perspectives from ethics, nursing, philosophy, psychology, mental health, and social science through concept mapping and examination under a rigorous data analytical framework of biostatistics and machine learning.

There is a promising potential for digital platforms to aid in the real-time identification of factors that mediate the potential for long-term psychological trauma and impairment associated with ethical conflicts. However, this will require significant modifications to the application of existing digital technologies and seamless integration across modalities. Integration with wearables and examination in virtual environments represent the next wave of digital innovation to understand patterns of physiological response and the development of machine learning training models, which would assist in predicting and responding to risks associated with moral distress.

This project is built upon the original proof-of-concept study, where a process and content evaluation of the technology platform, VR environment, and remote interventions will be undertaken by each participant over approximately 3 months. This will set the stage for more definitive studies to develop the potential of these technologies to predict and prevent moral distress. This project has the potential to develop innovative paradigms, which can be further examined in other occupational contexts prone to moral distress or injury (eg, military populations).

## Appendix

Multimedia Appendix 1 Educational video on stress and moral distress.

Multimedia Appendix 2 Moral Injury Outcome Scale (MIOS) versions used in this study.

Multimedia Appendix 3 Post-VR debrief interview guide.

Multimedia Appendix 4 Exit interview guide.

## Abbreviations

DPP: digital phenotype profile
EDA: electrodermal activity
GAD-7: Generalized Anxiety Disorder-7
HCW: health care worker
HRV: heart rate variability
MIOS: Moral Injury Outcome Scale
pDPP: personal digital phenotype profile
PHQ-9: Patient Health Questionnaire-9
PTSD: posttraumatic stress disorder
SID: statistics, information theory, and data-driven
SUDS: Subjective Units of Distress Scale
UCLA-3: UCLA 3-item Loneliness Scale
VR: virtual reality
VRET: VR exposure therapy
