# Supplementary material for: Digital Interventions to Understand and Mitigate Stress Response: Protocol for Process and Content Evaluation of a Cohort Study
Source: JMIR Res Protoc. 2024 May 6;13:e54180. doi: 10.2196/54180 (PMC11106701; doi:10.2196/54180)
Supplement: Multimedia Appendix 2 [file resprot_v13i1e54180_app2.docx]

Appendix 2: MIOS versions used in this study

**Brief MIOS**

**Moral Injury Outcome Scale**

**This questionnaire asks about experiences you may have had after a very stressful experience in which you *as a healthcare provider*: (A) did something (or failed to do something) that went against your moral code or values; or (B) you saw someone (or people) do something or fail to do something that went against your moral code or values; or (C) you were directly affected by someone (or systems) doing something or failing to do something that went against your moral code or values (e.g., being betrayed by someone you trusted).**

**Have you had at least one experience like this that troubles you currently? ___Yes ___No**

**If yes, please check the type of experience that is most currently distressing: ___A ___B ___C If more than one, check all that apply.**

**If you feel comfortable, please briefly describe this worst morally distressing event:**

**Keeping this experience in mind, please indicate how much you agree with the following statements in terms of the impact of this experience *in the last month* (circle one number for each item below).**

|  | ***Strongly***  ***Disagree*** | ***Disagree*** | ***Neither Agree or Disagree*** | ***Agree*** | ***Strongly***  ***Agree*** |
| --- | --- | --- | --- | --- | --- |
| **1. I blame myself.** | **0** | **1** | **2** | **3** | **4** |
| **2. I have lost faith in humanity.** | **0** | **1** | **2** | **3** | **4** |
| **3. People would hate me if they really knew me.** | **0** | **1** | **2** | **3** | **4** |
| **4. I have trouble seeing goodness in others.** | **0** | **1** | **2** | **3** | **4** |
| **5. People don’t deserve second chances.** | **0** | **1** | **2** | **3** | **4** |
| **6. I am disgusted by what happened.** | **0** | **1** | **2** | **3** | **4** |
| **7. I feel like I don’t deserve a good life.** | **0** | **1** | **2** | **3** | **4** |
| **8. I keep myself from having success.** | **0** | **1** | **2** | **3** | **4** |
| **9. I no longer believe there is a higher power.** | **0** | **1** | **2** | **3** | **4** |
| **10. I lost trust in others.** | **0** | **1** | **2** | **3** | **4** |
| **11. I am angry all the time.** | **0** | **1** | **2** | **3** | **4** |
| **12. I am not the good person I thought I was.** | **0** | **1** | **2** | **3** | **4** |
| **13. I have lost pride in myself.** | **0** | **1** | **2** | **3** | **4** |
| **14. I cannot be honest with other people.** | **0** | **1** | **2** | **3** | **4** |

**How much has this experience made it hard for you to function in each of the following areas (circle one number for each item below)? If an area is not applicable, circle N/A:**

|  | ***Not at all Somewhat Extremely*** | | | | | | |  |
| --- | --- | --- | --- | --- | --- | --- | --- | --- |
| **1. Romantic relationships with spouse or partner** | **0** | **1** | **2** | **3** | **4** | **5** | **6** | **N/A** |
| **2. Relationships with your children** | **0** | **1** | **2** | **3** | **4** | **5** | **6** | **N/A** |
| **3. Relationships with other family members** | **0** | **1** | **2** | **3** | **4** | **5** | **6** | **N/A** |
| **4. Friendships or socializing** | **0** | **1** | **2** | **3** | **4** | **5** | **6** | **N/A** |
| **5. Confidence, comfort, and competence in healthcare roles** | **0** | **1** | **2** | **3** | **4** | **5** | **6** | **N/A** |
| **6. Training or education** | **0** | **1** | **2** | **3** | **4** | **5** | **6** | **N/A** |
| **7. Day to day activities, such as chores, errands, managing medical care** | **0** | **1** | **2** | **3** | **4** | **5** | **6** | **N/A** |
| **8. Religious faith/spirituality** | **0** | **1** | **2** | **3** | **4** | **5** | **6** | **N/A** |

**MIOS-4**

1. **When completed during the virtual reality scenario: What is your personal reaction right now to the virtual scene?**
2. **When completed outside the virtual reality scenario: Right now, how strongly would you agree with the following statements:**

|  | ***Strongly Disagree*** | ***Disagree*** | ***Neither***  ***Agree or***  ***Disagree*** | ***Agree*** | ***Strongly Agree*** |
| --- | --- | --- | --- | --- | --- |
| **1. I feel shame.** | **0** | **1** | **2** | **3** | **4** |
| **2. I have lost faith in humanity.** | **0** | **1** | **2** | **3** | **4** |
| **3. I lost trust in others.** | **0** | **1** | **2** | **3** | **4** |
| **4. I am not the good person I thought I was.** | **0** | **1** | **2** | **3** | **4** |
