# Supplementary material for: Digital Interventions to Understand and Mitigate Stress Response: Protocol for Process and Content Evaluation of a Cohort Study
Source: JMIR Res Protoc. 2024 May 6;13:e54180. doi: 10.2196/54180 (PMC11106701; doi:10.2196/54180)
Supplement: Multimedia Appendix 3 [file resprot_v13i1e54180_app3.docx]

Appendix 3: Post-VR debrief interview guide

## Post-VR

- Thank you for participating. Are you ok to move forward to the debrief?
- The debrief will take approximately 15-20 minutes and we will keep the physiological sensors on until the end of the debrief. Is everything feeling okay?
- The debrief will start with me asking you about your feelings now vs. during the VR simulation, the thought process behind some of the decisions you made and then we’ll discuss the techniques you learned about during the intervention video. At the end of the debrief, we’ll have you complete three post-survey questions and give you the opportunity to provide us with some open-ended feedback about the VR.
- Throughout the debrief, we will use this voice recorder to record our conversation and my colleague will take note of your responses. She may interject during our conversation as well to ask you some questions about some things that stand out to her.
- Do you have any questions? Are you okay to proceed?

## Debrief

- The goal of the debrief is to understand the decisions you made during the scenario and the feelings associated with those decisions.
- We will show you some screenshots of the scenario so that you will have the opportunity to reflect on your actions and share your thinking process.
- Does this sound okay?

**Participants will take part in a semi-structured interview where they will answer the scenario checkpoints (i.e., screenshots) and questions in an open-ended manner. We will ask the following open-ended questions:*

Introduction & Participant Reactions:

- How are you feeling right now?

**Allow the participant to speak freely, if they are quiet, try prompting with further questions like the following*:*

- What emotions are you feeling?
- Can you tell me a bit more about why you are feeling this way?

Description

- Before we get further into the conversation, we want to quickly review what you have just experienced. To recap the scenario, you were one of the nurses in a medicine ward. One patient went into cardiac arrest, while was experiencing V-fib. You were forced to decide how to manage both emergencies simultaneously without much help.
- Is that what you recall? *Or:* Is that accurate with what you experienced?

Analysis – VR Scenario

- What emotions did you feel in the VR scenario?
- Did you feel any stress in the VR?
- *If they say yes,* Can you tell me a little more about how that felt? Have you felt this type of stress before? Can you describe that situation to me?
- Did this scenario violate your values or your beliefs in any way?

**If the participant does not understand or asks for further details, proceed with questions such as the following:*

- - Do you think the scenario went against your sense of right or wrong?
  - For example, did you feel guilty? What about shame? And anger or betrayal?
    - If the participant says no to the above feelings, then prompt with: Can you tell me a little bit about what emotions you did feel?

**Have 3-4 checkpoints chosen to discuss with participants. For the checkpoints at each screenshot, w*e should pose the question as follows:

“You were asked [insert question from VR here]. Can you elaborate on your perspective?”

- In the same video, we discussed techniques to cope with distress? Do you remember what these were?

*Depending on whether participants recall any or all of the interventions, ask them to describe what the intervention is (if they spontaneously recall the name) or remind them of the name and ask if they remember what it is?*

*If they do not recall all/specific interventions, remind them what they were.*

Before I clarify any definitions, can you rank on a scale of 1 to 5 how well you feel you understand each technique, with 1 being “I don’t understand at all” and 5 being “I understand perfectly.”

- In the video, we discussed three interventions: diaphragmatic breathing, unburdening, and self-compassion.

State:

1. Diaphragmatic breathing: on a scale of 1 to 5, how well do you understand this technique?

State definition if participant does not understand the technique: Diaphragmatic breathing involves taking deep breaths, counting to four as you breathe in, holding your breath for a count of four, breathing out for a count of four, and holding for four again. You can repeat this until you feel calmer.

1. Unburdening: on a scale of 1 to 5, how well do you understand this technique?

State definition if participant does not understand the technique: Unburdening involves speaking regularly with a trusted individual whom you can share your thoughts and feelings with and discuss any moral distress that you may be experiencing. The goal is to be vulnerable and open up, so you do not keep these feelings to yourself.

1. Self-compassion: on a scale of 1 to 5, how well do you understand this technique?

State definition if participant does not understand the technique: Self-compassion involves being kind in the way that you speak to yourself and treat yourself as you would treat a close friend who is struggling. In doing this, you remind yourself that we are all humans, and we all make mistakes.

**Debrief Summary:**

- Could you share something that you have learned about stress, in general and moral distress, in particular today? How might this apply to your clinical practice?
- Were any of the techniques discussed new to you? Which one was the most helpful? (*e.g., grounding, diaphragmatic breathing, unburdening, self-compassion).*
- In a few words, are you able to describe what you understand moral distress to be and what you remember about it?

Transition to Debrief Summary:

- Is there anything else that you were hoping to talk about before we wrap up?

**Transition:**

- This is the end of the debrief. Now, we will continue with your feedback on our experiment. We will ask you a few questions about the VR scenario and a quick survey with 3 questions about the experiment today. Any feedback you can provide is greatly appreciated and will help us improve the scenario and interventions in the future.
- Before we move onto these questions, we would like to confirm that you have the web platform (Greenspace) account set up. **If not, help them set it up**
- If you need help or have any questions about these platforms going forward, you can contact the research coordinator (Walter Sim).

**Open-ended feedback about the study:**

- What suggestions or feedback would you give to improve the scenarios?
- Is there anything that can be more realistic?

**Post survey questions**

1. I have learned a lot about moral distress and interventions

| Strongly disagree | Disagree | Neutral | Agree | Strongly agree |
| --- | --- | --- | --- | --- |
| 1 | 2 | 3 | 4 | 5 |

2. Knowledge of moral distress and intervention will help me perform better in real-life events

| Strongly disagree | Disagree | Neutral | Agree | Strongly agree |
| --- | --- | --- | --- | --- |
| 1 | 2 | 3 | 4 | 5 |

3. The emotions I experienced today felt the same as real-life

| Strongly disagree | Disagree | Neutral | Agree | Strongly agree |
| --- | --- | --- | --- | --- |
| 1 | 2 | 3 | 4 | 5 |

# 
