# Supplementary material for: Digital Interventions to Understand and Mitigate Stress Response: Protocol for Process and Content Evaluation of a Cohort Study
Source: JMIR Res Protoc. 2024 May 6;13:e54180. doi: 10.2196/54180 (PMC11106701; doi:10.2196/54180)
Supplement: Multimedia Appendix 4 [file resprot_v13i1e54180_app4.docx]

#### Appendix 4: Exit interview guide

#### **Introduction**

Hi, how are you?

My name is ____ and I am one of the team members conducting the study you have been taking part in with the Interventional Psychiatry Program.

Researcher: I understand that you are coming to the end of this study. First, I want to thank you so much for your participation and for the information you have provided us. As the final step of this study, I would like to ask you a few questions about your experience with the digital platforms and the interventions that we introduced to you. This will take approximately 30 minutes.

I will be recording this interview over Zoom. Is that okay?

**[Questions about distress experience with the simulation and similar feelings since then]**

We’ll start with some questions about your experience participating in the VR component of this study. You participated in a VR simulation where you assumed the role of a nurse working in a general medicine ward during the COVID-19 pandemic.

Can you briefly describe what you remember about this simulated experience?

When you think about the simulation, what feelings do you remember experiencing in the moment?

How do you feel about it currently, after three months? Is this different from how you felt at the time?

Over the last 3 months, have you felt any stress associated with the VR experience?

Have you found yourself thinking about the situation you were placed in over the last three months?

Have you experienced a situation that felt similar to how you felt during the simulation experience?

If you can explain, what was this experience? How did it feel?

**[Retention questions]**

If you recall, we also showed you an educational video about distress, moral distress, and techniques to manage this experience during the simulation. I want to ask you what you remember from this video, and if it has been useful to you.

In a few words, are you able to describe what you understand moral distress to be and what you remember about it?

Since you began participation in this study, do you feel you have experienced distress? Has this been in your job as a nursing professional?

Do you feel that an awareness of distress and moral distress has improved your experience at work? If yes, how? Has it changed how you view your experiences as a nurse?

In the same video, we discussed techniques to cope with distress? Do you remember what these were?

*Depending on whether participants recall any or all of the interventions, ask them to describe what the intervention is (if they spontaneously recall the name) or remind them of the name and ask if they remember what it is?*

*If they do not recall all/specific interventions, remind them what they were.*

In the video, we discussed three interventions: diaphragmatic breathing, unburdening, and self-compassion.

Before I clarify any definitions, can you rank, on a scale of 1 to 5 how well you feel you understand each technique, with 1 being “ I don’t understand at all” and 5 being “I understand perfectly.”

State:

1. Diaphragmatic breathing: on a scale of 1 to 5, how well do you understand this technique?

State definition if participant does not understand the technique:

Diaphragmatic breathing involves taking deep breaths, counting to four as you breathe in, holding your breath for a count of four, breathing out for a count of four, and holding for fouragain. You can repeat this until you feel calmer.

1. Unburdening: on a scale of 1 to 5, how well do you understand this technique?

State definition if participant does not understand the technique:

Unburdening involves speaking regularly with a trusted individual whom you can share your thoughts and feelings with and discuss any moral distress that you may be experiencing. The goal is to be vulnerable and open up, so you do not keep these feelings to yourself.

1. Self-compassion: on a scale of 1 to 5, how well do you understand this technique?

State definition if participant does not understand the technique:

Self-compassion involves being kind in the way that you speak to yourself and treating yourself as you would treat a close friend who is struggling. In doing this, you remind yourself that we are all humans and we all make mistakes.

**[Usefulness of interventions questions]**

Have you used any of these in difficult times at work over the last three months?

If yes, have you found them helpful? Which ones?

Have you tried to implement them and experienced any difficulties?

Like we did with your understanding, can you rank on a scale of 1 to 5 how helpful you feel each of these techniques were over the past three months, with 1 being not helpful at all and 5 being extremely helpful.

State:

1. Diaphragmatic breathing: on a scale of 1 to 5, how helpful was this technique?
2. Unburdening: on a scale of 1 to 5, how helpful was this technique?
3. Self-compassion: on a scale of 1 to 5, how helpful was this technique?

**[Experience with Oura and Web Platform]**

You have also been using the Oura Ring over the last three months. What was your experience like on this platform?

You have also been using a web-based platform over the last three months. What was your experience like with this platform?

Were there any specific barriers to use for either of these platforms that you found?

Did you find either or both platforms helped you in understanding your mental state and well-being over the past few months?

Thank you so much for your feedback on these platforms. We’re always looking to incorporate user experiences in order to improve the way we conduct the digital elements of our studies.

We also have two quick forms where we will ask you to describe your experience with the web-based platform and Oura Ring in terms of their similarity to a variety of words. For example, you would rank the Oura Ring on a scale of 1 to 7, where 1 and 7 are creative and dull. I’ve sent you two documents to fill one out - one for the Oura Ring and one for the web-based platform. It shouldn’t take more than a couple of minutes. Can you please fill these out and then send them back to the same email? I will stay on the line until you finish the forms to be sure you do not have any questions.

Okay, that is all the questions I have. I want to thank you again for your participation in this study and the feedback you have provided. Is there any other feedback, questions, or things you wanted to discuss about your experience as a participant in this research study?

This takes us to the end of the exit interview. You are no longer required to wear the ring or complete the questionnaires. Thank you once again for participating in this study. We wish you all the best.

[Interviewer’s subjective opinion based on what they said during the exit interview]: On a scale of 1-5, what was my sense of how well the experience went for the participant? Jot down any particularities about this participant.
